# Identifying Intersecting Factors Associated With Suicidal Thoughts and Behaviors Among Transgender and Gender Diverse Adults: Preliminary Conditional Inference Tree Analysis

**DOI:** 10.2196/65452

**Published:** 2025-04-11

**Authors:** Amelia M Stanton, Lauren A Trichtinger, Norik Kirakosian, Simon M Li, Katherine E Kabel, Kiyan Irani, Alexandra H Bettis, Conall O’Cleirigh, Richard T Liu, Qimin Liu

**Affiliations:** 1 Department of Psychological and Brain Sciences Boston University Boston, MA United States; 2 Fenway Health Boston, MA United States; 3 Division of Mathematics, Computing, and Statistics Simmons University Boston, MA United States; 4 Department of Psychology University of Miami Coral Gables, FL United States; 5 Department of Psychiatry and Behavioral Sciences Vanderbilt University Medical Center Nashville, TN United States; 6 Department of Psychiatry Massachusetts General Hospital Boston, MA United States; 7 Department of Psychiatry Harvard Medical School Boston, MA United States

**Keywords:** transgender and gender diverse adults, suicidality, intersectionality, conditional inference tree, electronic medical record

## Abstract

**Background:**

Transgender and gender diverse (TGD) individuals are disproportionately impacted by suicidal thoughts and behaviors (STBs), and intersecting demographic and psychosocial factors may contribute to STB disparities.

**Objective:**

We aimed to identify intersecting factors associated with increased risk for suicidal ideation, intent, plan, and attempts in the US transgender population health survey (N=274), and determine age of onset for each outcome using conditional inference trees (CITs), which iteratively partitions subgroups of greater homogeneity with respect to a specific outcome.

**Methods:**

In separate analyses, we restricted variables to those typically available within electronic medical records (EMRs) and then included variables not typically within EMRs. We also compared the results of the CIT analyses with logistic regressions and Cox proportional hazards models.

**Results:**

In restricted analyses, younger adults endorsed more frequent ideation and planning. Adults aged ≤26 years who identified as Black or with another race not listed had the highest risk for ideation, followed by White, Latine, or multiracial adults aged ≤39 years who identified as sexual minority individuals. Adults aged ≤39 years who identified as sexual minority individuals had the highest risk for suicide planning. Increased risk for suicidal intent was observed among those who identified as multiracial, whereas no variables were associated with previous suicide attempts. In EMR-specific analyses, age of onset for ideation and attempts were associated with gender identity, such that transgender women were older compared to transgender men and nonbinary adults when they first experienced ideation; for attempts, transgender women and nonbinary adults were older than transgender men. In expanded analyses, including additional psychosocial variables, psychiatric distress was associated with increased risk for ideation, intent, and planning. High distress combined with high health care stereotype threat was linked to increased risk for intent and for suicide planning. Only high everyday discrimination was associated with increased risk for lifetime attempts. Ages of onset were associated with gender identity for ideation, the intersection of psychiatric distress and drug use for suicide planning, and gender identity alone for suicide attempts. No factors were associated with age of onset for suicide intent in the expanded variable set. The results of the CIT analysis and the traditional regressions were comparable for ordinal outcomes, but CITs substantially outperformed the regressions for the age of onset outcomes.

**Conclusions:**

In this preliminary test of the CIT approach to identify subgroups of TGD adults with increased STB risk, the risk was primarily influenced by age, racial identity, and sexual minority identity, as well as psychiatric distress, health care stereotype threat, and discrimination. Identifying intersecting factors linked to STBs is vital for early risk detection among TGD individuals. This approach should be tested on a larger scale using EMR data to facilitate service provision to TGD individuals at increased risk for STBs.

## Introduction

### Background

Suicide is a leading cause of death in the United States [1], and transgender and gender diverse (TGD) individuals experience heightened risk for suicidal thoughts and behaviors (STBs). Lifetime prevalence of suicide attempts is 40% among gender minority individuals [2], relative to 4% in the general population [3]. In a recent analysis of Danish national hospital records, standardized suicide attempt rates per 100,000 person years were 498 for transgender individuals compared to 71 among individuals who were not transgender [4]. These disparities align with documented disparities in other areas, in that TGD individuals are at significantly greater risk for experiencing other mental health issues (eg, depression and anxiety) [5-7], substance use disorders [8-10], bullying [11-13], and sexual abuse or intimate partner violence [14-16] in comparison to their cisgender peers.

Suicide disparities in TGD individuals are likely driven by multiple factors associated with marginalization (eg, discrimination, internalized stigma, and associated depression) [17,18] in combination with factors known to drive STBs in general samples (eg, financial stress, unemployment, relationship problems, and physical health problems) [19-21] and other suicide-specific theoretical drivers (eg, thwarted belongingness and perceived burdensomeness) [22]. In the recent past, the marginalization of TGD persons has become increasingly systematized. From 2018 to 2022, 48 laws restricting the rights of transgender and nonbinary individuals were enacted across the United States; in the states that enacted those laws, reports of past-year suicide attempts among TGD youth increased by 7% to 72% [23]. Minority stress theory suggests that individuals who hold stigmatized identities, across domains, experience disproportionately high stress that results from that stigma [11,24,25]. Within this framework, stigma-related stressors may be external to the self (eg, harassment) or experienced internally because of consistent exposure to societal stigma (eg, negative attitudes toward the self). TGD individuals bear a long history of experiencing societal discrimination and oppression, as well as substantial disparities in mental health outcomes [26], likely the result of cisnormativity (ie, the assumption or expectation that all people are cisgender, or have gender identities that align with their sex assigned at birth [27-29]) in society, and the prejudice associated with cisgenderism, which then lead to bias and discrimination, harassment and violence, rejection and misgendering, and associated internal stressors (eg, gender dysphoria, internalized transphobia, or transnegativity) [30,31]. Several studies have demonstrated that the key pathways articulated via minority stress theory have strong empirical support among TGD populations [32-34]. TGD populations are also subject to the factors that drive STB risk in the general population as well as the psychological and interpersonal factors that are highlighted in established theoretical models of suicidality, including the interpersonal theory of suicide [22]. Recent research has integrated these theoretical models to suggest that the intersection of minority stressors and general suicide theoretical precursors drives STB risk in TGD people [35].

Emerging evidence also suggests that individuals exposed to intersectional forms of marginalization (eg, transphobia and racism) may have unique experiences relative to individuals with one marginalized identity, and that these unique experiences may lead to even worse health outcomes. Originally developed to describe the unique intersection of racism and sexism in the United States [36-39], intersectionality theory simultaneously accounts for multiple forms of marginalization, investigates the social processes that perpetuate inequity, and explores the meaning of living in an intersectional position. Intersectional minority stress for multiply marginalized individuals may start in childhood. With continued discrimination and stigmatization across contexts, intersectional minority stress persists and accumulates alongside adulthood stressors and distress, both of which are associated with suicide risk [40].

Data-driven approaches to quantifying intersectionality have the potential to precisely identify groups that may have elevated risks of suicidal ideation (SI) and factors associated with suicide-related outcomes [41,42]. Researchers have called for more sophisticated and targeted statistical methods for studying intersectionality [43], especially to explore intersecting sociodemographic factors beyond the common *big three* identifiers of race, gender, and socioeconomic status [41,44]. One such approach, known as the conditional inference tree [45], iteratively partitions samples into subgroups of greater homogeneity with respect to a specific outcome. Compared to mixture modeling (eg, latent profile analysis for continuous data and latent class analysis for categorical data), the conditional inference tree allows a more realistic representation of multivariate data due to its ability to approximate complex distributions and relations and to detect heterogeneity specific to an outcome. Conditional inference trees can also be more advantageous than conventional linear models, which focus on linear relationships only and often fail to account for the ways in which multiple factors interact in complex and nonlinear fashions to influence outcomes [46]. The conditional inference tree approach allows for the characterization of distinct, empirically derived “profiles” or subgroups, characterized by the presence of co-occurring factors that together predict increased STB risk.

### Objectives

With potential applications for health systems in mind, this study tests the conditional inference tree approach to identify the intersecting factors that characterize subpopulations of TGD adults who are at increased risk for 4 different STBs (SI, intent, plan, and history of previous attempts). We report 2 sets of analyses: one that restricts variables to those that are typically available within an electronic medical record (EMR; eg, age, gender identity, ethnoracial identity, sexual orientation, and public assistance status) and one that expands the set of variables to include urbanicity and psychosocial factors that are not commonly available within EMRs (eg, discrimination, psychiatric distress, gender minority stress, alcohol use, drug use, social well-being, and health care stereotype threat) but have demonstrated associations with STBs among TGD individuals. Discriminatory events are predictors of suicidal self-injury in this population [47], and TGD individuals who have experienced gender-based discrimination are 4 times more likely to have attempted suicide than those who have not [48]. Both psychiatric distress (ie, experiencing distress associated with psychological disorders), which is more prevalent among TGD individuals than cisgender individuals [49], and gender minority stress are associated with increased SI and behaviors [50-53]. Substance use, especially in the context of psychiatric distress or depression, has been associated with increased odds of STBs among transgender youth [54]. Social well-being is likely also linked to STBs among TGD individuals [55], and new data suggests that health care stereotype threat has a significant direct, adverse association with self-rated health and psychological distress among gender minority individuals [56], which may have implications for STBs.

Novel quantitative approaches for assessing intersectionality are necessary to examine (1) how sociodemographic and psychosocial factors are experienced in combination (ie, how do demographic and psychosocial factors interact) and (2) how sociodemographic factors operate within socialized hierarchies and health systems (ie, which factors are most associated with STBs). Given that inequities and disparities associated with different demographic and psychosocial factors often combine to exacerbate negative health outcomes, identifying the intersecting factors associated with STBs will help elucidate how and where TGD individuals are situated in socialized hierarchies and systems. By leveraging data that are typically available in most EMRs, the first set of analyses will offer an initial test of the data-driven, conditional inference tree approach to identify TGD individuals that may need to be prioritized for additional risk assessment, appropriate resources, and treatment referrals in health systems or other clinical settings. The second set of analyses will offer more nuanced information on subgroups of TGD persons at increased risk for STBs and may inform the selection of measures that could be integrated into EMRs.

## Methods

### Participants

We included 274 TGD participants from the US transgender population health survey (TransPop), a national probability sample of gender diverse adults in the United States that was conducted from 2016 to 2018, which is publicly available [57]. Probability sampling approaches were used to enhance diversity and representativeness of the sample [58] (see Krueger et al [59] for further methodological details on the original study). In brief, the TransPop study was the first large-scale, national probability sample of TGD individuals in the United States, designed to provide comprehensive data on the social, economic, physical, and mental health experiences of TGD individuals. Probability sampling approaches were used to enhance diversity and representativeness of the sample [58] through the Gallup Daily Tracking Survey. In total, 2 recruitment methods were used as follows: (1) random digit dialing, to reach both cellphone and landline users and (2) address-based sampling. Respondents were asked about their sex assigned at birth and their gender; those who reported male sex assigned at birth and identified their gender as “woman,” those who reported female sex assigned at birth and identified their gender as “man,” and those who identified as “transgender” were defined as transgender. Respondents were also screened for other eligibility requirements: adult aged ≥18 years, education above the sixth grade level, and ability to complete an interview in English. In addition, cisgender participants were included as a comparison group to examine disparities; however, the cisgender sample was not analyzed in this study.

It is important to highlight that the TransPop study was a cross-sectional examination of the psychosocial experiences of TGD individuals. As such, all variables were assessed at the same time point, with measures that probed various time frames; this enabled us to use current variables to assess associations with past outcomes. Though these relationships will need to be assessed prospectively in large, longitudinal datasets, this dataset offered us the opportunity to explore a novel use for conditional inference trees, with hopes that this approach and other data-driven techniques may identify subgroups of TGD individuals that may benefit from enhanced screening or early, prospective intervention.

### Ethical Considerations

The TransPop study protocol was reviewed and approved by the Gallup Institutional Review Board (IRB), the University of California, Los Angeles IRB, and the IRBs of collaborating institutions through reliance on University of California, Los Angeles IRB. Collaborating institutions included Columbia University; University of Texas at Austin; University of California, Santa Cruz; University of California, San Francisco; University of Arizona; University of Surrey, United Kingdom; and University College London, United Kingdom. When invited to participate, potential respondents were sent a US $25 Amazon gift card (if invited via email) or US $25 in cash (if invited via mail). Participants were asked to review an information sheet before beginning the survey, and their consent was assumed if they completed the questions and submitted the survey to the researchers. Participants were therefore not asked to sign consent forms because the relying IRB determined that a signed consent document would impose an unnecessary risk to participant confidentiality. Identifying data were separated from study data and kept confidential at Gallup; investigators did not have access to identifying data at any time.

For additional details on ethical considerations, please refer to the methodology section and technical notes document that was prepared by the principal investigators [59].

### Measures

#### Sociodemographic Variables

We considered the associations between 7 sociodemographic variables and STBs: age, ethnoracial identity, gender identity, sexual minority status, urbanicity, public assistance status, and personal income. The study included participants from five ethnoracial groups: White (non-Hispanic); Black or African American; Hispanic, Latino, or Spanish origin; multiracial (ie, individuals who selected more than one racial and ethnic category); and other (ie, individuals who identified as Middle Eastern or North African, Native Hawaiian or Pacific Islander, or American Indian or Alaskan Native). Gender identity included 3 categories: transgender man, transgender woman, and gender queer or nonbinary. Sexual minority status was a binary variable indicating the presence or absence of a minoritized sexual identity. Urbanicity (urban vs nonurban) was computed using respondents’ zip codes based on the United States Department of Agriculture rural-urban commuting area coding system. Public assistance status indicated receipt (1 vs 0) of the Supplemental Nutrition Assistance Program or the Special Supplemental Nutrition Program for Women, Infants, and Children. Personal income (per year) was rated from no income to ≥US $150,000 in US $5000 increments.

#### Psychological and Clinical Variables

We also considered 7 psychosocial variables: alcohol use, drug use, gender minority stress, experiences of discrimination, distress, social well-being, and health care stereotype threat.

##### Alcohol Use

Alcohol use was measured using the Alcohol Use Disorders Identification Test-Concise, a 3-item scale designed to identify persons with hazardous drinking behavior, or who have active alcohol use disorders [60]. Items include monthly alcohol consumption frequency, daily alcohol consumption frequency, and binge drinking frequency. Each item was rated on a 5-point Likert scale from 0 to 4, and individual item scores were summed to create a total score, with higher scores indicating that the individual’s alcohol use is negatively affecting their health and safety. Reliability analysis indicated high internal consistency for this measure (Ω=0.88). The Alcohol Use Disorders Identification Test has been determined to be reliable among transgender adults [61].

##### Drug Use

Drug use was measured via the Drug Use Disorders Identification Test, an 11-item scale designed to identify individuals with drug-related problems or substance use disorders [62]. Each item was rated on a 5-point Likert scale from 0 to 4. The final variable was the sum of all variables in the scale, and a higher score indicated greater substance use. Reliability analysis indicated high internal consistency for this measure (Ω=0.96). To our knowledge, the Drug Use Disorders Identification Test does not appear to have been validated for use among transgender adults.

##### Psychiatric Distress

The Kessler-6 was used to assess psychiatric distress [63]. Notably, the Kessler-6 primarily focuses on negative affect, anxiety, and depression symptoms rather than other forms of emotional distress (such as anger, which has been linked to suicide risk [64]). Scale items measured the frequency of the following emotions or experiences in the past 30 days: “nervous,” “hopeless,” “restless or fidgety,” “so depressed that nothing could cheer you up,” “that everything was an effort,” and “worthless.” Responses were recorded on a 5-point scale, ranging from “all of the time” to “none of the time.” All items were first reverse-coded so that “none of the time” had a value of 1 and “all of the time” had a value of 5. The final score was the sum of all individual item scores. Reliability analysis indicated high internal consistency for this measure (Ω=0.93). The Kessler-6 does not appear to be validated specifically among transgender adults but has been used widely in this population [65-67].

#### Variables Related to Gender Minority Identity

##### Gender Minority Stress

Gender minority stress was measured with the following four subscales of the Gender Minority Stress and Resilience measure [31]: (1) *Internalized transphobia* (eg, “I resent my transgender identity,” and “I ask myself why I can’t just be normal?”) measures the degree to which individuals have internalized or integrated societal stigma into their own self-concepts (Ω=0.90), (2) *Non-affirmation of gender identity* (eg, “I have to repeatedly explain my gender identity to people or correct the pronouns people use,” and “I have difficulty being perceived as my gender”) assesses the degree to which individuals feel that their gender identity is understood and accepted by others (Ω=0.95), (3) *Nondisclosure of gender identity* (eg, “I don’t talk about certain experiences from my past or I change parts of what I will tell people,” and “I modify my way of speaking”) measures the degree to which individuals avoid disclosing their gender identity to others (Ω=0.81), and (4) *Negative expectations of the future* (eg, “if I express my gender identity/history, others wouldn’t accept me,” and “if I express my gender identity/history, employers would not hire me”) assesses the degree to which an individual believes that they will not be understood or accepted because of their gender identity in the future (Ω=0.93). Responses were recorded on 5-point Likert scales, ranging from “strongly disagree” to “strongly agree.” The mean scores of each subscale, which ranged from 1 to 5, were used in analyses.

##### Discrimination

The Everyday Discrimination Scale was used to assess daily experiences of discrimination or unfair treatment [68]. For example, scale items probe how often the following experiences occurred over the past year: “You were treated with less courtesy than other people,” “You were treated with less respect than other people,” and “You were called names or insulted.” Responses were recorded on a 4-point Likert scale, ranging from “often” to “never.” Scale values ranged from 1 to 4. The final score was the mean of all items. Higher values represent more everyday discrimination. Reliability analysis indicated high internal consistency for this measure (Ω=0.95). The Everyday Discrimination Scale has demonstrated partial metric invariance across transgender and cisgender groups, and within gender identities among transgender respondents [69]. The Trans Discrimination Scale was developed and published in 2019 [70], after the TransPop study was executed.

##### Health Care Stereotype Threat

A modified 4-item version of the scale developed by Abdou and Fingerhut [71] was used to assess the degree to which participants worried about being negatively judged by their health care providers or confirming stereotypes about lesbian, gay, bisexual, and transgender people in health care settings (eg, “I worry about being negatively judged because of my sexual orientation or gender identity”). Responses were recorded on a 5-point scale, ranging from “strongly disagree” to “strongly agree.” The mean score across all items was used in subsequent analyses, with lower values representing less worry about being judged or confirming lesbian, gay, bisexual, and transgender stereotypes and higher values representing greater worry. Reliability analysis indicated high internal consistency for this measure (Ω=0.93). Although the scale has been used previously in transgender samples [72], it has not been psychometrically validated in this population.

#### Variables Related to Well-Being: Social Well-Being

Social well-being is defined as one’s “appraisal of one’s circumstances and functioning in society” [73]. The social well-being scale, developed by Keyes, that was included in the TransPop survey consists of 15 items (eg, “I don’t feel I belong to anything I’d call a community,” “My community is a source of comfort,” and “I have something valuable to give to the world”), each rated on a 7-point Likert scale ranging from “strongly disagree” to “strongly agree.” The mean score across all items was used; higher values represent greater social well-being. Final scores ranged from 1 to 7. Reliability analysis indicated high internal consistency for this measure (Ω=0.85). To our knowledge, this scale has not been specifically validated in transgender populations.

#### Primary Outcomes

We assessed 4 types of STBs, which were measured by participants’ responses to the questions in parentheses as follows: (1) *SI* (“Did you ever in your life have thoughts of killing yourself?”), (2) *suicidal intent* (“Did you ever have any intention to act on thoughts of wishing you were dead or trying to kill yourself?”), (3) *suicide plan* (“Did you ever think about how you might kill yourself, e.g., taking pills, shooting yourself, or work out a plan of how to kill yourself?”), and (4) *suicide attempt history* (“Did you ever make a suicide attempt, i.e., purposefully hurt yourself with at least some intention to die?”). Respondents rated each of the 4 STBs as “No,” “Yes, once,” or “Yes, more than once.” In addition, if a participant endorsed a given outcome, they were additionally asked to provide the best estimate for the age of first onset of that outcome (“how old were you the very first time you...”).

### Statistical Analysis

We applied conditional inferences trees [45] using the partykit package in R (R Foundation for Statistical Computing) to identify subgroups with intersecting demographic and psychosocial factors that are associated with increased likelihood of each of the 4 suicidal outcomes. Conditional inference trees model the nonlinear relationships between a wide range of predictors and an outcome. As a data mining approach, the conditional inference tree is a data-driven analytic strategy that identifies interacting social determinants from many candidate predictors to determine which predictors are most relevant to specific outcomes. Conventionally, researchers have used generalized and general linear models with interaction terms, as informed by theory, to model intersectionality; these approaches are limited in that only a small number of predictors are typically examined simultaneously and confined by assumed additive and linear effects, and they require follow-up tests (eg, Tukey’s tests) to determine actionable groups that deserve additional attention [74,75]. Importantly, conditional inference trees can highlight potential statistical predictors for between-group differences (eg, poverty as an additional intersectional factor for younger individuals experiencing SI). This is advantageous for intersectionality research, because our goal is not only to uncover subgroups that explain the heterogeneity in STBs but also to understand the *factors* associated with the heterogeneity. Finally, conditional inference tree can effectively handle smaller sample sizes, as methodological research has shown reliable results with subgroup sizes as small as 10 to 20 participants [76].

We conducted 2 sets of analyses. First, we used variables that approximate basic data that may be collected in EMRs, with the understanding that health systems vary, as do the data that are typically collated in these records. These variables included age, gender identity, ethnoracial identity, sexual minority status, and public assistance status. This list was based on data that is consistently collected from patients within a large academic health system in the northeastern United States and patients receiving care from a community health center, also in the northeastern United States, with which study authors are affiliated. Age was not included as a predictor for the age of onset analyses. Second, we included the following additional psychosocial variables in the models: personal income, urbanicity, alcohol use, drug use, psychiatric distress, specific constructs related to gender minority stress (internalized transphobia, nonaffirmation of gender identity, nondisclosure of gender identity, and negative expectations of the future), discrimination, health care stereotype threat, and social well-being. It is important to note that some of the variables included in the second set of analyses are sometimes collected in EMRs (eg, in one report, 40% of patients had alcohol use documented in their EMRs) [77], but the demographic factors specified as variables in the first set may be more consistently available. For each set of analyses, we used conditional inference trees to examine the ordinal lifetime history of each of the 4 STBs, as well as age of first onset for those outcomes.

For the outcomes specific to lifetime history of the 4 STBs, all models were trained using 10-fold cross-validation with the caret package in R. If the *P* value was less than the criterion before reaching maximum depth, which were selected after being treated as hyperparameters in the model, a node split was implemented [45]. The model with the largest accuracy value was selected. The maximum depth ranged from 1 to 5 with the deepest tree observed for suicidal plan with the EMR variables. The *P* value criterion ranged from .001 to .05, with the strictest thresholds used in the models with suicidal intent and suicide attempt with the EMR variables.

For models examining factors associated with age of onset of the 4 STBs, a 10-fold cross-validation approach was used to tune the conditional inference trees by optimizing the same 2 hyperparameters (a list of all hyperparameters is provided in Tables S1 and S2 in Multimedia Appendix 1). The optimal combination was identified based on the lowest root mean square error (RMSE) across all folds to balance predictive performance and model complexity. The maximum depth was predominantly selected to be 1 or 2 except for the suicide plan model with the non-EMR variables. The *P* value criterion was generally consistent at .05 with 2 stricter thresholds for suicide plan with EMR variables and suicidal intention with non-EMR variables. All variables had <1% missing data, except for sexual minority identity, which had 1.1% missingness. Missing data were handled using surrogate splits.

To compare results from the conditional inference trees with those of more traditional regression analyses, we conducted ordinal regression and Cox proportional hazard models as alternatives. However, there are fundamental differences between these approaches: regression models provide a global view of predictor effects, while conditional inference trees reveal complex interactions and nonlinear relationships, rendering them particularly effective in addressing intersectional research questions. Results are detailed in Tables S1-S4 in Multimedia Appendix 1.

This study was conducted and reported in accordance with the STROBE (Strengthening the Reporting of Observational Studies in Epidemiology) guidelines for cross-sectional studies [78]. A completed STROBE checklist is provided in Multimedia Appendix 2.

## Results

### Prevalence of STBs

In this sample (N=274), 80.3% (n=220) of participants endorsed SI, 54.7% (n=150) endorsed suicidal intent, 67.2% (n=184) endorsed having a suicide plan, and 36.1% (n=99) endorsed a history of suicide attempts, with 49 (17.9%) participants endorsing 1 previous suicide attempt and 50 (18.2%) participants endorsing >1 previous attempts. Table 1 provides participant demographics. Table 2 displays further descriptive statistics of study variables.

**Table 1:** Participant demographics.

			Full sample		Transgender men		Transgender women		Nonbinary adults
Gender identity, n (%)			274 (100)		78 (28.47)		120 (43.8)		76 (27.74)
Age (y), mean (SD)			39.36 (16.89)		34.71 (15.92)		46.18 (16.71)		33.38 (14.23)
Sexual minority identity, n (%)			213 (78.6)		51 (65.38)		88 (75.21)		74 (97.37)
Urbanicity, n (%)			217 (79.2)		64 (82.05)		89 (74.17)		64 (84.21)
**Race, n (%)**									
	White	187 (68.25)		50 (64.1)		85 (70.83)		52 (68.42)	
	Black	21 (7.66)		8 (10.26)		8 (6.67)		5 (6.58)	
	Latino	26 (9.49)		7 (8.97)		10 (8.33)		9 (11.84)	
	Multiracial	24 (8.76)		8 (10.26)		10 (8.33)		6 (7.89)	
	Other	16 (5.84)		5 (6.41)		7 (5.83)		4 (5.26)	
**Personal income (US $), n (%)**									
	No income	16 (5.84)		4 (5.13)		6 (5)		6 (7.89)	
	1-4999	27 (9.85)		13 (16.67)		6 (5)		8 (10.53)	
	5000-9999	33 (12.04)		8 (10.26)		18 (15)		7 (9.21)	
	10,000-14,999	29 (10.58)		10 (12.82)		8 (6.67)		11 (14.47)	
	15,000-19,999	30 (10.95)		8 (10.26)		14 (11.67)		8 (10.53)	
	20,000-24,999	16 (5.84)		5 (6.41)		7 (5.83)		4 (5.26)	
	25,000-29,999	13 (4.74)		6 (7.69)		5 (4.17)		2 (2.63)	
	30,000-39,999	21 (7.66)		6 (7.69)		8 (6.67)		7 (9.21)	
	40,000-49,999	20 (7.3)		4 (5.13)		8 (6.67)		8 (10.53)	
	50,000-59,999	19 (6.93)		6 (7.69)		8 (6.67)		5 (6.58)	
	60,000-74,999	10 (3.65)		2 (2.56)		5 (4.17)		3 (3.95)	
	75,000-99,999	16 (5.84)		2 (2.56)		12 (10)		2 (2.63)	
	100,000-149,999	15 (5.47)		3 (3.85)		10 (8.33)		2 (2.63)	
	≥150,000	9 (3.28)		1 (1.28)		5 (4.17)		3 (3.95)	

**Table 2:** Descriptive statistics of study variables.

			Score ranges		Full sample (N=274)		Transgender men (n=78)		Transgender women (n=120)		Gender nonbinary adults (n=76)
Alcohol use, mean (SD)			0-12		2.14 (2.04)		1.77 (1.84)		2.13 (2.13)		2.53 (2.02)
Drug use, mean (SD)			0-44		3.95 (6.27)		3.18 (5.41)		3.38 (5.89)		5.64 (7.36)
Psychiatric distress, mean (SD)			0-23		9.26 (5.85)		8.59 (5.54)		8.7 (6.18)		10.83 (5.38)
Everyday discrimination, mean (SD)			1-4		2.07 (0.79)		2.00 (0.78)		2.03 (0.82)		2.2 (0.75)
Social well-being, mean (SD)			1-7		4.46 (0.95)		4.46 (0.85)		4.52 (0.93)		4.37 (1.08)
Health care stereotype threat, mean (SD)			1-5		3.28 (1.18)		3.37 (1.11)		3.04 (1.28)		3.59 (0.99)
Nonaffirmation of gender identity, mean (SD)			1-5		2.98 (1.25)		2.60 (1.35)		2.75 (1.19)		3.74 (0.89)
Gender identity nondisclosure, mean (SD)			1-5		3.39 (0.91)		3.47 (0.88)		3.41 (0.93)		3.27 (0.9)
Internalized transphobia, mean (SD)			1-5		2.64 (1.01)		2.74 (0.98)		2.69 (1.02)		2.46 (1)
Negative expectations of the future, mean (SD)			1-5		3.18 (0.92)		3.08 (0.96)		3.11 (0.94)		3.38 (0.80)
**Lifetime suicidal ideation, n (%)**											
	No	—^a^		53 (19.41)		14 (18.18)		30 (25)		9 (11.84)	
	Yes, once	—		43 (15.75)		9 (11.69)		24 (20)		10 (13.16)	
	Yes, more than once	—		177 (64.84)		54 (70.13)		66 (55)		57 (75.00)	
**Lifetime suicidal intent, n (%)**											
	No	—		123 (45.05)		30 (38.96)		67 (55.83)		26 (34.21)	
	Yes, once	—		53 (19.41)		17 (22.08)		18 (15)		18 (23.68)	
	Yes, more than once	—		97 (35.53)		30 (38.96)		35 (29.17)		32 (42.11)	
**Lifetime suicide plan, n (%)**											
	No	—		89 (32.6)		20 (25.97)		48 (40)		21 (27.63)	
	Yes, once	—		48 (17.58)		15 (19.48)		22 (18.33)		11 (14.47)	
	Yes, more than once	—		136 (49.82)		42 (54.55)		50 (41.67)		44 (57.89)	
**Lifetime suicide attempt, n (%)**											
	No	—		174 (63.74)		45 (58.44)		81 (67.5)		48 (63.16)	
	Yes, once	—		49 (17.95)		17 (22.08)		21 (17.5)		11 (14.47)	
	Yes, more than once	—		50 (18.32)		15 (19.48)		18 (15)		17 (22.37)	

^a^Not applicable.

### EMR-Specific Variables Associated With STBs

**Figure 1:**
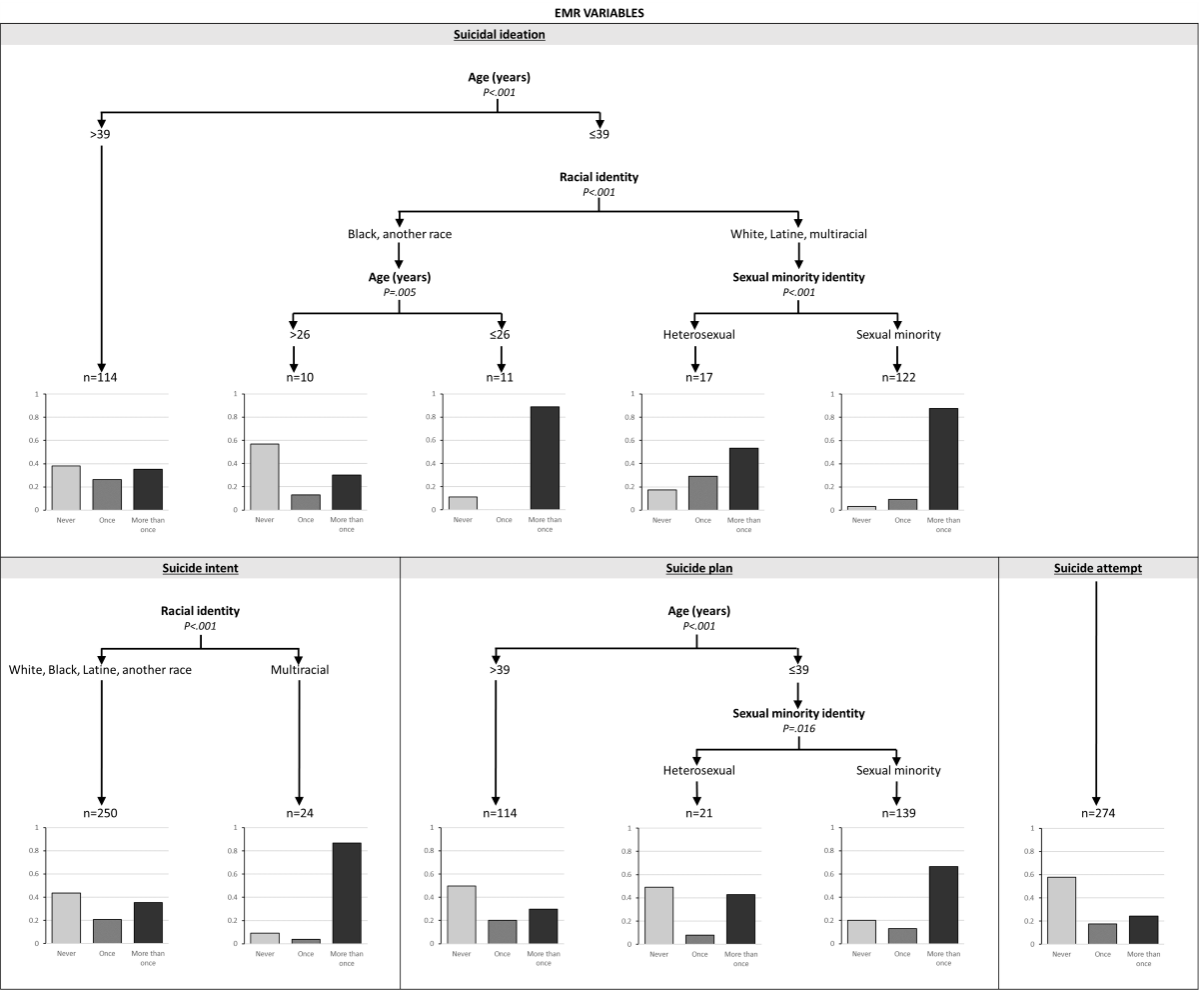
Tree diagram for electronic medical record (EMR)–specific variables predicting lifetime suicidal ideation, intent, planning, and attempts. each decision tree groups participants by EMR variables. Terminal nodes show response distributions (No, Yes once, Yes more than once), with numbers above nodes indicating cases and percentages representing subgroup proportions. Numbers above nodes indicate participants with history of the outcome, while percentages below represent subgroup proportions.

Figure 1 presents the co-occurring EMR-specific variables (age, gender identity, ethnoracial identity, sexual minority status, and public assistance status) that were associated with lifetime history of SI, suicidal intent, suicide plans, and suicide attempts.

For SI, age emerged as a significant primary factor (*P*<.001). For younger participants (aged ≤39 years), racial identity emerged as a secondary factor. For young White, Latine, and multiracial participants, the model identified sexual minority identity as tertiary factor, such that those who identified as sexual minority individuals were more likely to endorse lifetime history of SI. Young Black participants and participants who identified with another race were further split based on age. Younger participants (aged ≤26 years) were more likely to endorse lifetime history of SI than those aged >26 years. The model had a modest accuracy of 60.4%. For suicidal intent, race emerged as the sole differentiating factor. Participants who identified as multiracial had an increased likelihood of suicidal intent. The model accuracy was 47.6%. For suicide plan, age again emerged as the primary factor. For younger participants (aged ≤39 years), sexual minority identity was a secondary factor, such that individuals aged ≤39 years who had sexual minority identities were more likely to have a suicide plan. The model had an accuracy of 55%. Importantly, no EMR-specific variables were associated with a history of suicide attempts.

**Figure 2:**
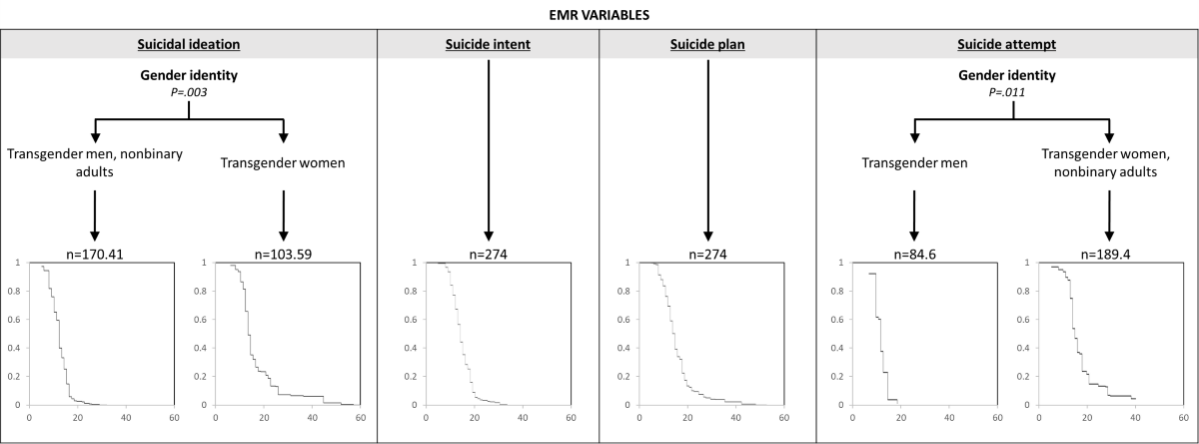
Survival diagrams for electronic medical record (EMR)–related variables predicting age of onset for suicidal ideation, suicide intent, suicide plan, and suicide attempt. Each decision tree groups participants by EMR variables, with terminal nodes displaying survival curves showing the probability of no history of each outcome.

Figure 2 presents the intersection of EMR-specific variables that were associated with age of onset for SI, intent, plan, and history of previous attempts. With respect to SI, age of onset was meaningfully and primarily differentiated by gender identity, such that transgender women (median=14 years) were older than nonbinary individuals and transgender men (median=12 years) when they first experienced SI. The model had an RMSE of 9.04. No EMR-related variables were associated with age of onset for suicidal intent or plan. For a history of suicide attempts, transgender women and nonbinary individuals (median=15 years) were older than transgender men (median=13 years) when they first experienced a suicide attempt. The model had an RMSE of 7.24.

### Including Additional Psychosocial Variables to Examine Associations With STBs

**Figure 3:**
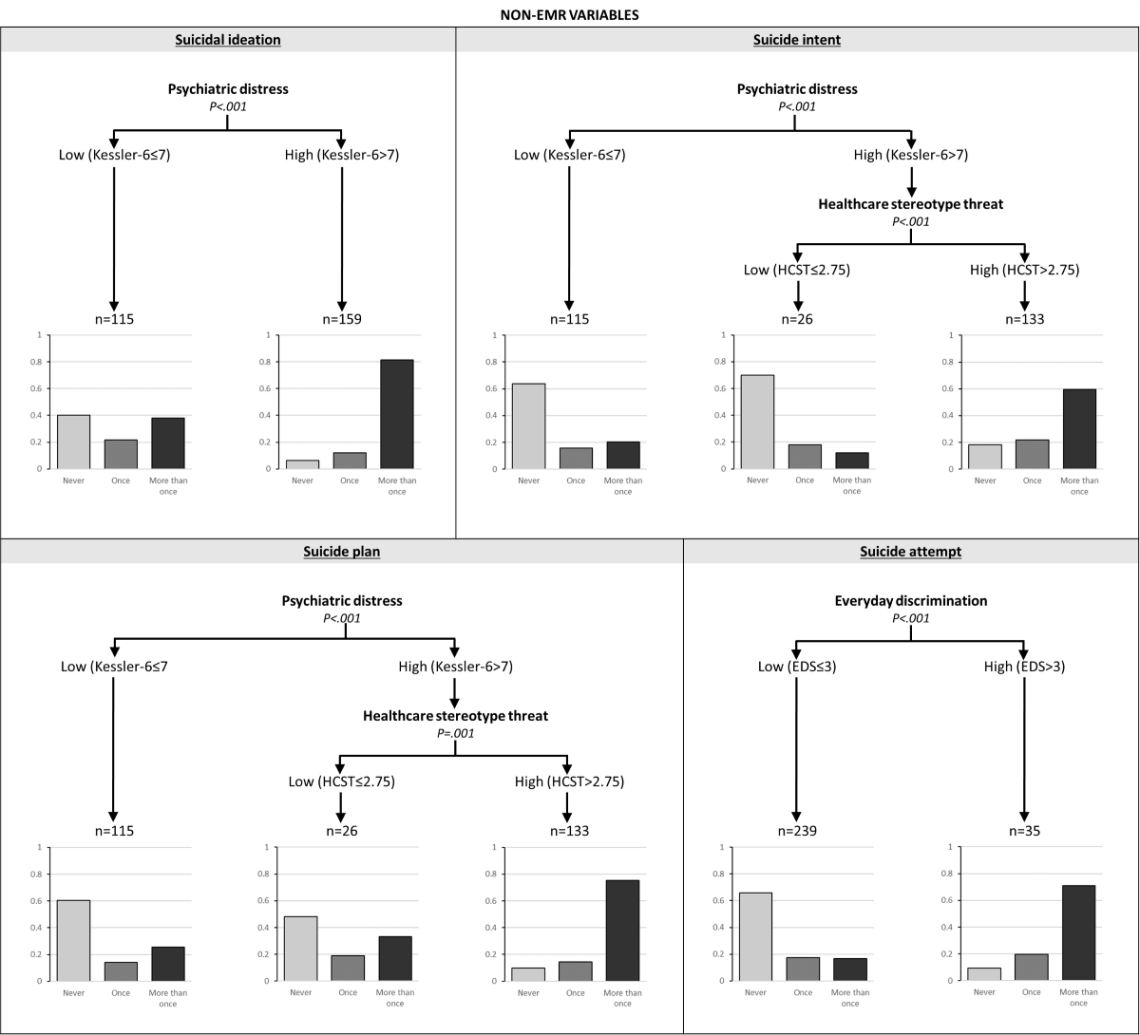
Tree diagrams for the expanded set of variables predicting lifetime history of suicidal ideation, suicide intent, suicide plan, and suicide attempt. Each decision tree groups participants by electronic medical record–specific and psychosocial variables. Terminal nodes show response distributions (No, Yes once, Yes more than once), with percentages indicating the proportion of each subgroup reporting any history of the outcome. EDS: Everyday Discrimination Scale; EMR: electronic medical record; HCST: health care stereotype threat.

Figure 3 depicts the intersection of variables that were associated with a lifetime history of the 4 STBs. To conduct this analysis, all variables (EMR-specific variables combined with urbanicity and the additional psychosocial variables) were added to the models and examined. Psychiatric distress was consistently demonstrated to be the primary differentiating factor for SI, intent, and plan; that is, adults with higher psychiatric distress were more likely to endorse a lifetime history of all 3 types of suicidal thinking (ideation, intent, and planning). Health care stereotype threat emerged as a consistent secondary factor for identifying persons at increased risk for suicidal intent and plan. Among TGD adults experiencing higher psychiatric distress, those who had lower health care stereotype threat had lower odds of suicidal intent or suicide plan. For a history of previous suicide attempts, experiencing higher levels of discrimination was the sole primary differentiating factor, with higher discrimination positively associated with an increased likelihood of attempts. The models for SI, intent, plan, and attempts had accuracy levels of 60.8%, 56.8%, 60.4%, and 68.5%, respectively.

**Figure 4:**
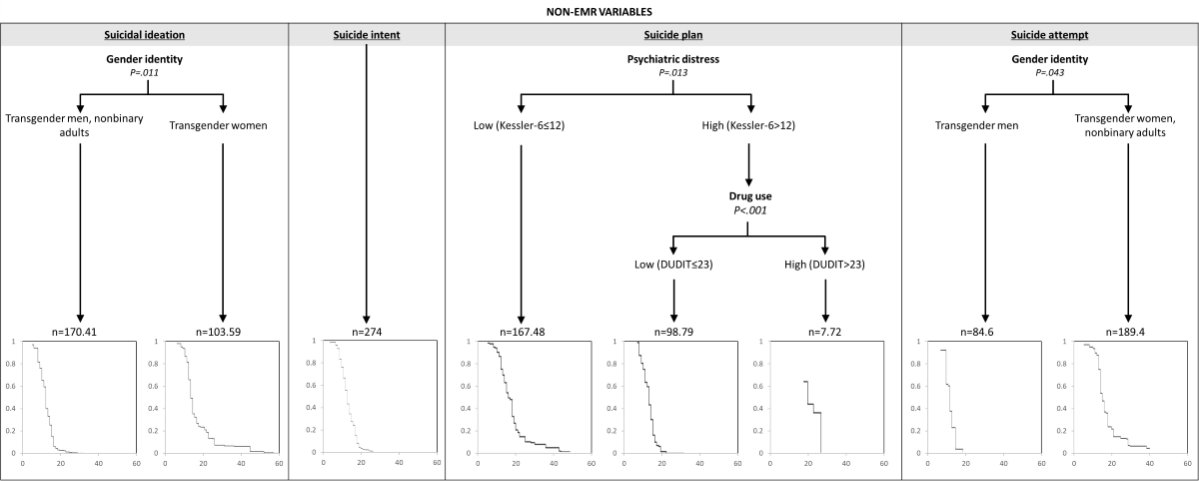
Tree diagrams for the expanded set of variables predicting age of onset for suicidal ideation, suicide intent, suicide plan, and suicide attempt. Each decision tree groups participants by electronic medical record–specific and psychosocial variables. Each terminal node displays the survival curve indicating the probability of participants in that subgroup not having a history of each outcome. DUDIT: Drug Use Disorders Identification Test; EMR: electronic medical record.

Figure 4 presents the intersection of all included variables that were associated with age of onset for each of the 4 suicide outcomes. For SI, transgender women (median=14 years) had substantially later age of onset than nonbinary individuals and transgender men (median=12 years; *P*=.01). The RMSE was 9.04. For suicide intent, age of onset did not differ by included variables. With respect to suicide plans, adults with both high psychiatric distress and high drug use had the latest age of onset of planning for suicide (median=21.5 years). Adults with high psychiatric distress but low drug use had the earliest age of onset (median=14 years), and adults with low psychiatric distress had a median age of onset of 15 years. The model RMSE was 8.24. For suicide attempts, transgender women and nonbinary individuals (median=15 years) had significantly later age of onset than transgender men (median=13 years; *P*=.04). The model RMSE was 7.24.

The logistic regressions and Cox proportional hazards models corroborated the relevance and significance of most of the variables selected by the conditional inference trees. In terms of performance, the conditional inference tree approach and the more traditional regression methods were comparable for ordinal outcomes (Tree model accuracy=.48-.69; logistic accuracy=.53-.70). However, the conditional inference trees substantially outperformed the regressions for the age of onset outcomes (Tree RMSE=5.73-9.04; Cox proportional hazards RMSE=16.24-20.32). The results of these additional analyses are available in Multimedia Appendix 1.

## Discussion

### Principal Findings

In this population-based sample of TGD adults, we identified that the following intersecting factors are associated with at least one of the 4 STBs: age, racial identity, sexual minority identity, psychiatric distress, health care stereotype threat, and everyday discrimination. In addition, across both the limited set of variables intended to approximate data available in an EMR and the expanded set of variables that included additional psychosocial constructs, analyses revealed that gender identity, psychiatric distress, and drug use were associated with age of onset of the 4 STBs. Isolating the intersectional factors that are associated with increased risk for STBs, as well as the ages of onset for those outcomes, is an important step in identifying individuals who may need additional support and selecting points of intervention, especially at the health systems level. Most importantly, we demonstrate that conditional inference trees are a viable, data-driven strategy for capturing intersecting risk factors, suggesting that, when used in larger samples, this approach may identify clinically meaningful subgroups of TGD adults or other patient populations who are at increased risk for STBs.

When we restricted the variables to data that may be available within EMRs, age, racial identity, and sexual orientation played key roles. Specifically, younger age and Black or another racial identity as well as the combination of younger age; White, Latine, and multiracial identities; and sexual minority status were associated with increased risk for SI. Multiracial identity was the primary and only factor associated with increased risk for suicidal intent but was not associated with suicide planning, for which young age and minority sexual orientation conferred the most risk. None of the variables in the restricted dataset were associated with previous suicide attempts. In a large, representative sample (>250,000 respondents) of the general US population, pulled from the National Survey on Drug Use and Health, past-year prevalence of suicide-related thoughts and planning was higher among adults between the ages of 18 and 39 years than among those aged ≥40 years [79]. Age-related differences in SI have also been documented across cultures, including in a South Korean sample [80], in which young adults were more likely than older adults to experience significant SI, regardless of depression severity. It is possible that younger individuals may feel more directly impacted by the current sociopolitical climate and associated restrictions on access to gender-affirming care across the country. The intersection of younger age and minority racial identity [81] as well as the intersection of younger age and sexual minority status has been associated with increased risk for STBs in other samples [82], though not to this degree of specificity; risk is often compared between TGD and cisgender individuals rather than within TGD populations. While traditional EMRs capture key demographic variables, they often lack crucial psychosocial factors that may convey important information about STB risk. These findings suggest that, when only limited data are available, subgroups of TGD individuals with increased risk for STBs can still be identified.

When additional psychosocial variables not typically contained within EMRs were added to the models, recent psychiatric distress was the differentiating factor for suicidal thoughts (ideation, intent, and plan), with health care stereotype threat emerging as a secondary intersecting factor for intent and plan. Distress is a consistent predictor of STBs across contexts and populations [83,84]. In general, integrating self-reported data with EMR data appears to improve suicide risk prediction models [85]. Although we were able to identify demographic factors associated with increased risk of suicidal thoughts when the model included only the variables that approximated those typically found in EMRs, the addition of other relevant constructs for TGD adults, especially health care stereotype threat, offers unique insights on factors that could be assessed and intervened upon in health systems. Defined as the fear of confirming negative stereotypes by one’s group and the fear that one’s group status negatively influences how medical providers evaluate and diagnose patients [71], health care stereotype threat has been associated with increased anxiety, distrust of providers, and decreased adherence to health behaviors [71,86]. Importantly, patients for whom high health care stereotype threat is highly relevant to suicide intent or planning (ie, those with high distress) may be most likely to be missing or underrepresented in EMR assessments of risk; that is, health care stereotype threat may lead to disparities in receipt of needed care. Stigmatizing interactions with health care providers and institutions may have a particularly strong impact on health and well-being disparities [87-89]. In the 1 other study [56] that examined this construct among TGD adults, health care stereotype threat had a direct adverse association with self-rated health and psychological distress, even after accounting for experiencing discrimination and stigma. Though preliminary, these results suggest that integrating a brief measure assessing patient experiences with health care providers may help facilitate STB risk identification.

In addition, experiencing everyday discrimination was the only differentiating factor that was associated with increased risk for previous suicide attempts. There is a longstanding established association between discrimination and suicide deaths or history of suicide attempts among TGD individuals (Clements-Nolle et al [90]) that has also been confirmed in recent studies. For example, in an Australian study of TGD adults conducted in 2021, institutional discrimination (ie, from employment, housing, accessing health care, or government services) related to their gender identity was positively associated with a history of suicide attempts [91]. Among TGD adults in the current sample, those who experienced everyday discrimination were most likely to endorse a history of suicide attempts. Similarly, when free-text clinical notes from medical visits that took place before suicide attempts were examined, over half had evidence of being misgendered in the health care system, and at times, patient reports of being misgendered within the health system were directly documented in the notes [92]. These instances of bias and discrimination in health care settings may further discourage TGD patients from disclosing SI [93] and from seeking mental health care, even when they are at acute risk of suicide. If the pattern observed in these data is replicated, everyday discrimination may be a key differentiator for suicide attempts over different forms of suicidal thinking (ie, ideation, intent, and planning) because it acts as a cumulative, acute stressor that directly impacts behavior through pathways involving stress escalation, acquired capability, and acute psychological pain [22,25,94]. While ideation reflects prolonged distress and contemplation, attempts often result from acute events or provocations such as discrimination, particularly when coping resources are insufficient. Future studies should consider exploring systematic strategies to identify TGD patients who have had discriminatory experiences in health care or elsewhere, as they may benefit from enhanced screening or suicide prevention resources.

Age of onset for SI and attempts was associated with gender identity alone in the EMR-specific analyses, with no variables emerging for suicidal intent and plan; however, when additional variables were added into the models, the intersection of distress and drug use provided insights on risk for suicide planning. Transgender women were older than nonbinary individuals and transgender men when they first experienced SI, and transgender women and nonbinary individuals were older than transgender when they first experienced a suicide attempt. Adults with both high psychiatric distress and high drug use had the latest age of onset of planning for suicide, whereas adults with high psychiatric distress and low drug use had the earliest age of onset. To our knowledge, no other studies have identified intersectional factors that predict age of onset for STBs among TGD individuals. From a health systems perspective, it may be critical to identify the approximate ages at which STBs emerge, particularly among subgroups at increased risk for earlier onset (eg, transgender men and nonbinary individuals, and individuals with high psychiatric distress).

### Limitations and Future Directions

Several limitations of this study warrant mention and point to important future directions for use of the conditional inference tree approach in suicidality research among TGD persons and other populations at increased risk. First, the small sample size may preclude identification of important factors that had weak or variable effects, as the statistical power to detect such effects is limited in a smaller dataset; this may also have impacted the robustness of subgroup identification. Despite this limitation, the use of conditional inference trees is a robust approach for smaller samples, as it effectively handles complex interactions and avoids overfitting through unbiased variable selection. This allows us to identify and interpret the most significant relationships in the data, even with a relatively small sample. Nonetheless, subsequent studies should, seek to use larger samples pulled from EMRs to identify more nuanced subgroups within health symptoms that may be at increased risk for STBs. Similarly, despite a larger set of factors associated with STBs that were considered in this study compared to previous research, consideration of other factors beyond demographic or psychosocial variables may aid in identification of subgroups who are at elevated risk of STBs, should such variables be accessible within larger datasets. In addition, because this study used variables to approximate those common to EMRs, replication of our findings with data that have actually been extracted from health systems across regions is important to evaluate the appropriateness and clinical applicability of the conditional inference tree approach for use with EMR data. Notably, the authors’ affiliations with community health clinics and large academic medical centers located in the northeastern United States may have led to the selection of variables that approximate data available in EMRs within this region, and not across other regions of the United States or in other countries. In addition, the variables selected here were intended to approximate data available within these records to demonstrate the potential of the conditional inference tree approach to identify high risk subgroups within large systems. As Streed et al [95] articulated, sexual orientation and gender identity data have not historically been collected in EMRs, despite the high relevance of this information for the provision of high-quality clinical care. However, per Streed et al [95], efforts to draw attention to this critical gap have led to data systems changes and, in 2016, a requirement by the Health Resources and Service Administration’s Bureau of Primary Health Care to collect and provide sexual orientation and gender identity data in all federally funded community health centers. Hopefully, these changes will continue such that these data elements will be uniformly captured across EMR platforms. While the use of a national probability sample of TGD adults enhances generalizability, it is important to consider potential underreporting of STBs in this population. Underreporting of suicidality may be more common in this population due to health care stereotype threat or other forms of discrimination and marginalization [93,96,97]. In addition, the categorization of sexual orientation (heterosexual vs sexual minority individuals) may have masked additional risk experienced by bisexual individuals, among whom relatively high levels of STBs have been documented [98-100].

Finally, 2 substantial limitations of these analyses are the cross-sectional design of the survey and the varied time frames of assessments. STBs are highly dynamic and episodic, with studies demonstrating quick onset and relatively short duration of suicidal thoughts [101,102], such that capturing ideation in a single cross-sectional survey is challenging. Moreover, variables included in the models were assessed at different time points (eg, current gender minority stress, past 30-day psychiatric symptoms, past-year experiences of discrimination, and lifetime suicidal STBs), such that almost all psychosocial factors assessed are current or recent, whereas outcomes are measured at across the lifetime or related to age of onset. Given that most individuals have SI onsets in adolescence [103], limited conclusions can be drawn from these associations. Rather than a conclusive determination of the specific subgroups of TGD individuals who may be at increased risk for STBs, these data offer strong preliminary evidence that this methodology can be leveraged in larger samples to potentially yield clinically meaningful results.

### Conclusions

In conclusion, we applied a novel data mining technique to isolate intersecting factors associated with STBs, as well as their respective ages of onset, in a national probability sample of TGD adults. Although there have been advances in transgender health care (eg, insurance coverage for gender-affirming care, bias trainings for providers, and the establishment of treatment and care standards) [104-107], there has also been a large movement across the United States to restrict access to affirming care, which may contribute to increased risk for marginalization, discrimination, associated minority stress, and suicidality [23]. Importantly, we have demonstrated the viability of the conditional inference tree approach in isolating subgroups of TGD adults who are at increased risk for STBs. If these associations are confirmed in larger, prospective studies that leverage health systems data, risk detection and service provision can be enhanced. Ultimately, the ability to identify persons with intersecting risk factors within health systems will support the deployment of data-enhanced screening and multilevel suicide risk reduction interventions that are affirming and comprehensive.

## Data Availability

The dataset analyzed during this study are available in the TransPop repository [108].

## Appendix

Multimedia Appendix 1 Comparison of conditional inference trees, ordinal regression models, and Cox proportional hazard models in predicting suicidal thoughts and behaviors.

Multimedia Appendix 2 STROBE (Strengthening the Reporting of Observational Studies in Epidemiology) checklist.

## Abbreviations

EMR: electronic medical record
IRB: Institutional Review Board
RMSE: root mean square error
SI: suicidal ideation
STB: suicidal thoughts and behavior
STROBE: Strengthening the Reporting of Observational Studies in Epidemiology
TGD: transgender and gender diverse
