# Supplementary material for: Identifying Intersecting Factors Associated With Suicidal Thoughts and Behaviors Among Transgender and Gender Diverse Adults: Preliminary Conditional Inference Tree Analysis
Source: J Med Internet Res. 2025 Apr 11;27:e65452. doi: 10.2196/65452 (PMC12032506; doi:10.2196/65452)
Supplement: Multimedia Appendix 1 [file jmir_v27i1e65452_app1.docx]

**Multimedia Appendix 1.** Comparison of conditional inference trees, ordinal regression models, and Cox proportional hazard models in predicting suicidal thoughts and behaviors.

To corroborate relevance of variables identified in the conditional inference trees, we additionally ran ordinal regression models for ordinal outcomes for suicidal thoughts and behaviors and cox proportional hazard models for age of onset for suicidal thoughts and behaviors. While regression models corroborated most variables identified in the conditional inference tree, some additional predictors emerged in the regression models that were not present in the conditional inference tree, and vice versa. It is worth noting that the fundamental difference in rationale and applications between these two methods likely accounts for the observed discrepancy. Conditional inference trees prioritize capturing complex, non-linear interactions and hierarchical relationships among predictors, making them particularly suited for exploring variable importance and interactions in a data-driven manner. In contrast, regression models, such as ordinal regression and Cox proportional hazard models, rely on predefined linear or parametric relationships between predictors and outcomes, allowing for hypothesis testing and quantifying effect sizes. This divergence reflects how each method approaches the underlying structure of the data: the conditional inference tree excels in uncovering nuanced patterns without strong parametric assumptions, while regression models focus on validating direct associations under specified assumptions. Together, these methods complement one another by offering both exploratory insights and confirmatory evidence, providing a more comprehensive understanding of the factors associated with suicidal thoughts and behaviors.

This discrepancy also highlights the importance of integrating approaches like conditional inference trees and regression models when modeling intersectionality and identifying intersectional subgroups or factors. Conditional inference trees are particularly valuable in intersectional research because they can reveal how combinations of variables—such as gender, sexual orientation, socioeconomic status, and mental health risk factors—interact to create unique subgroups associated with distinct outcomes. The conditional inference tree models align with the core tenets of intersectionality by capturing the non-additive and complex relationships between overlapping identities and systemic factors.

**Ordinal Regression Models with EMR variables**

The results from the EMR model highlight key demographic predictors across suicidal ideation, suicidal intent, suicidal plan, and suicide attempt. Age was a significant predictor in both tree and regression models for suicidal ideation (log(OR) = -0.06, 95% CI [-0.08, -0.04], *p* < 0.001) and suicide plan (log(OR) = -0.05, 95% CI [-0.07, -0.03], *p* < 0.001), indicating that older individuals had lower odds of experiencing these suicidal behaviors. However, age was only significant in the regression model for suicide intent (log(OR) = -0.04, 95% CI [-0.06, -0.02], *p* < 0.001) and suicide attempt (log(OR) = -0.03, 95% CI [-0.05, -0.01], *p* = 0.007). Race was a significant factor in both tree and regression models for suicidal ideation (*p* < 0.001) and suicide intent (*p* < 0.001), but only in regression for suicide plan (*p* < 0.001) and suicide attempt (*p* < 0.001). Sexual minority identity was significant in both models for suicidal ideation (log(OR) = 1.2, 95% CI [0.52, 1.9], *p* < 0.001) and suicide plan (log(OR) = 0.83, 95% CI [0.15, 1.5], *p* = 0.017). Public assistance was only significant in the regression model for suicide intent (*p* = 0.022) and suicide attempt (*p* = 0.001).

**Ordinal Regression Models with EMR and psychosocial variables**

Psychiatric distress was a significant predictor for both suicidal ideation (log(OR) = 0.19, 95% CI [0.11, 0.28], *p* < 0.001) and suicide plan (log(OR) = 0.15, 95% CI [0.08, 0.23], *p* < 0.001) in both models. healthcare stereotype threat was significantly associated with suicide intent in both models (log(OR) = 0.6, 95% CI [0.26, 0.96], *p* < 0.001), though it was only significant in the tree model for suicide plan and in the regression for suicide attempt (log(OR) = 0.64, 95% CI [0.29, 1.0], *p* < 0.001). Race was a significant predictor in regression for suicidal ideation (*p* = 0.040), suicide intent (*p* < 0.001), suicide plan (*p* = 0.017), and suicide attempt (*p* = 0.016), highlighting racial disparities in suicide risk. Sexual minority identity was only significant in regression for suicidal ideation (log(OR) = 0.93, 95% CI [0.04, 1.9], *p* = 0.040). Public assistance was significant only in regression for suicidal ideation (*p* = 0.028), as was personal income (log(OR) = 0.12, 95% CI [0.02, 0.22], *p* = 0.022). Social wellbeing was protective for suicidal ideation (log(OR) = 0.57, 95% CI [0.17, 0.99], *p* = 0.005), suicide plan (log(OR) = 0.36, 95% CI [0.01, 0.72], *p* = 0.046), and suicide attempt (log(OR) = 0.37, 95% CI [0.00, 0.75], *p* = 0.049) in the regression model only. Likewise, drug use was significant in regression for suicide intent (log(OR) = 0.06, 95% CI [0.01, 0.11], *p* = 0.023). Everyday discrimination significantly predicted suicide attempt in both models (log(OR) = 0.8, 95% CI [0.32, 1.3], *p* < 0.001), emphasizing the impact of discrimination on suicide attempts. Finally, non-affirmation of gender identity was significant in regression for suicide attempt (log(OR) = -0.35, 95% CI [-0.67, -0.03], *p* = 0.030).

**Cox Proportional Hazards with EMR variables**

The Cox proportional hazards model for the EMR variables identified key predictors. Gender identity was a significant predictor only in the tree for both suicidal ideation (*p* = 0.003) and suicide attempt (*p* = 0.011). Being a trans woman was associated with lower risk in the regression for suicidal ideation (HR = 0.44, 95% CI [0.26, 0.75], *p* = 0.002), suicide plan (HR = 0.51, 95% CI [0.30, 0.87], *p* = 0.013), and suicide attempt (HR = 0.20, 95% CI [0.06, 0.65], *p* = 0.008). Gender Non-binary (GNB) individuals also had lower risk for suicide attempt in the regression model (HR = 0.33, 95% CI [0.15, 0.71], *p* = 0.005). Individuals identifying as an “Other” racial identity had an increased risk for suicide attempt (HR = 6.26, 95% CI [1.98, 19.7], *p* = 0.002) in the regression only. Similarly, multiracial identity was significantly associated with increased risk for suicidal ideation (HR = 2.69, 95% CI [1.48, 4.90], *p* = 0.001) and suicide intent (HR = 4.31, 95% CI [2.16, 8.60], *p* < 0.001) in the regression, while Latino identity was a significant predictor of higher risk in the regression for suicide intent (HR = 2.54, 95% CI [1.40, 4.61], *p* = 0.002). Public assistance was a significant protective factor in the regression model for suicide plan (HR = 0.44, 95% CI [0.26, 0.73], *p* = 0.002).

**Cox Proportional Hazards with EMR and psychosocial variables**

The Cox proportional hazards models including both EMR and psychosocial variables identified several significant predictors. Drug use (HR = 0.93, 95% CI [0.90, 0.97], *p* < 0.001) and psychiatric distress (HR = 1.10, 95% CI [1.05, 1.15], *p* < 0.001) were significant in both the survival plot and regression models, reinforcing their strong association with suicide planning. Gender identity was a significant predictor in the survival plot model only for suicidal ideation (*p* = 0.011) and suicide attempt (*p* = 0.043). Relatedly, trans women showed a lower risk for suicidal ideation (HR = 0.38, 95% CI [0.21, 0.69], *p* = 0.002) and suicide plan (HR = 0.48, 95% CI [0.27, 0.85], *p* = 0.012) in the regression. Latinos had a higher risk in the regression for suicidal ideation (HR = 3.21, 95% CI [1.96, 5.25], *p* < 0.001) and suicide intent (HR = 2.25, 95% CI [1.15, 4.38], *p* < 0.018), while multiracial individuals were associated with higher suicidal ideation (HR = 3.11, 95% CI [1.52, 6.33], *p* = 0.002). Other racial groups were associated with higher risk in the regression for suicidal ideation (HR = 3.02, 95% CI [1.20, 7.63], *p* = 0.019), suicide plan (HR = 3.47, 95% CI [1.64, 7.35], *p* = 0.001), and suicide attempt (HR = 9.72, 95% CI [1.13, 83.7], *p* = 0.038). Additionally, urbanicity (HR = 0.50, 95% CI [0.31, 0.81], *p* = 0.005), alcohol use (HR = 0.80, 95% CI [0.71, 0.91], *p* < 0.001), psychiatric distress (HR = 1.07, 95% CI [1.02, 1.12], *p* = 0.009), and internalized transphobia (HR = 0.71, 95% CI [0.54, 0.93], *p* = 0.013) were significant in regression for suicidal ideation. Psychiatric distress (HR = 1.14, 95% CI [1.05, 1.25], *p* = 0.003), everyday discrimination (HR = 2.04, 95% CI [1.22, 3.41], *p* = 0.007), and social wellbeing (HR = 1.56, 95% CI [1.08, 2.26], *p* = 0.018) were significant only in regression for suicide intent. Public assistance was a protective factor for suicide intent (HR = 0.23, 95% CI [0.09, 0.58], *p* = 0.002) and suicide attempt (HR = 0.32, 95% CI [0.17, 0.60], *p* < 0.001). Everyday discrimination (HR = 1.44, 95% CI [1.05, 1.97], *p* = 0.023) and gender identity non-disclosure (HR = 1.40, 95% CI [1.09, 1.79], *p* = 0.008) were also significant only in regression for SP.

| **Table 1.** Hyperparameters with EMR variables. | | | |  |
| --- | --- | --- | --- | --- |
| Outcomes | STBs | | Age Onset | |
|  | Maxdepth | Criterion | Maxdepth | Criterion |
| Suicidal Ideation | 4 | 0.95 | 1 | 0.95 |
| Suicide Intent | 4 | 0.999 | 1 | 0.95 |
| Suicide Plan | 2 | 0.95 | 1 | 0.999 |
| Suicide Attempt | 3 | 0.999 | 2 | 0.95 |
| Note. The following hyperparameter values were tested for selecting the optimal value Maxdepth: 1,2,3,4,5 and Mincriterion: 0.95, 0.99, 0.995, 0.999, 0.9995, 0.9999 | | | | |
|  | | | |  |

| **Table 2.** Selected Hyperparameters with both EMR and psychosocial variables. | | | | | | | |  |
| --- | --- | --- | --- | --- | --- | --- | --- | --- |
| Outcomes | STBs | | | | Age Onset | | | |
|  | Maxdepth | | Criterion | | Maxdepth | | Criterion | |
| Suicidal Ideation | 1 | | 0.995 | | 1 | | 0.95 | |
| Suicide Intent | 2 | | 0.95 | | 1 | | 0.99 | |
| Suicide Plan | 5 | | 0.995 | | 4 | | 0.95 | |
| Suicide Attempt | 1 | | 0.95 | | 1 | | 0.95 | |
| Note. The following hyperparameter values were tested for selecting the optimal value Maxdepth: 1,2,3,4,5 and Mincriterion: 0.95, 0.99, 0.995, 0.999, 0.9995, 0.9999 | | | | | | | | |
|  | |  | |  | |  | |  |

| **Table 3.** Ordinal regression with EMR variables. | | |  |  |  |  |  |  |  |  |  |  |
| --- | --- | --- | --- | --- | --- | --- | --- | --- | --- | --- | --- | --- |
| Characteristics | Suicidal Ideation | | | Suicide Intent | | | Suicide Plan | | | Suicide Attempt | | |
|  | log(OR) | 95% CI | p-value | log(OR) | 95% CI | p-value | log(OR) | 95% CI | p-value | log(OR) | 95% CI | p-value |
| Age | **-0.06** | **[-0.08, -0.04]** | **<0.001** | **-0.04** | **[-0.06, -0.02]** | **<0.001** | **-0.05** | **[-0.07, -0.03]** | **<0.001** | **-0.03** | **[-0.05, -0.01]** | **0.007** |
| **Gender identity** |  |  | >0.9 |  |  | 0.100 |  |  | 0.800 |  |  | >0.9 |
| Trans Woman | 0.08 | [-0.62, 0.79] |  | 0.29 | [-0.33, 0.92] |  | 0.15 | [-0.49, 0.78] |  | 0 | [-0.62, 0.63] |  |
| GNB | 0.1 | [-0.67, 0.88] |  | 0.68 | [0.05, 1.3] |  | 0.18 | [-0.48, 0.84] |  | -0.12 | [-0.76, 0.52] |  |
| **Race** |  |  | **<0.001** |  |  | **<0.001** |  |  | **<0.001** |  |  | **<0.001** |
| Black | -1.3 | [-2.1, -0.37] |  | -0.17 | [-1.0, 0.67] |  | -0.64 | [-1.5, 0.20] |  | -0.26 | [-1.2, 0.62] |  |
| Latino | -0.02 | [-0.81, 0.80] |  | 0.58 | [-0.10, 1.3] |  | -0.12 | [-0.84, 0.60] |  | 0.37 | [-0.32, 1.1] |  |
| Multiracial | 1.5 | [0.27, 3.0] |  | 2.6 | [1.5, 3.9] |  | 2.1 | [0.96, 3.4] |  | 1.7 | [0.86, 2.5] |  |
| Other | -1.1 | [-2.1, 0.03] |  | -0.73 | [-1.8, 0.25] |  | -0.62 | [-1.6, 0.36] |  | -0.11 | [-1.2, 0.89] |  |
| Sexual minority identity | **1.2** | **[0.52, 1.9]** | **<0.001** | 0.38 | [-0.32, 1.1] | 0.300 | **0.83** | **[0.15, 1.5]** | **0.017** | 0.48 | [-0.23, 1.2] | 0.200 |
| **Public assistance** |  |  | 0.600 |  |  | **0.022** |  |  | 0.300 |  |  | **0.001** |
| Yes vs. No | -0.22 | [-0.98, 0.56] |  | 0.85 | [0.12, 1.6] |  | 0.4 | [-0.32, 1.1] |  | 1.1 | [0.45, 1.9] |  |
| Abbreviations. OR = Odds Ratio, CI = Confidence Interval, GNB = Gender Non-binary | | | | | | | | |  | | |  |
| Note. Reference category for gender identity was trans man and for race was White. | | | | | | | | |  |  |  |  |

| **Table 4.** Ordinal regression with both EMR and psychosocial variables. | | | | | |  |  |  |  |  |  |  |
| --- | --- | --- | --- | --- | --- | --- | --- | --- | --- | --- | --- | --- |
| Characteristics |  | Suicidal Ideation |  | Suicide Intent | | | Suicide Plan | | | Suicide Attempt | | |
|  | log(OR) | 95% CI | p-value | log(OR) | 95% CI | p-value | log(OR) | 95% CI | p-value | log(OR) | 95% CI | p-value |
| Age | -0.02 | [-0.05, 0.00] | 0.051 | 0.00 | [-0.03, 0.02] | 0.800 | -0.01 | [-0.04, 0.01] | 0.300 | 0.01 | [-0.02, 0.03] | 0.500 |
| **Gender identity** |  |  | 0.300 |  |  | 0.300 |  |  | 0.500 |  |  | 0.200 |
| Trans Woman | -0.54 | [-1.4, 0.30] |  | 0.00 | [-0.72, 0.72] |  | -0.4 | [-1.1, 0.34] |  | -0.63 | [-1.4, 0.12] |  |
| GNB | -0.74 | [-1.7, 0.24] |  | 0.54 | [-0.22, 1.3] |  | -0.33 | [-1.1, 0.47] |  | -0.26 | [-1.0, 0.53] |  |
| **Race** |  |  | **0.040** |  |  | **<0.001** |  |  | **0.017** |  |  | **0.016** |
| Black | -1.2 | [-2.3, -0.01] |  | -0.40 | [-1.4, 0.58] |  | -0.48 | [-1.5, 0.55] |  | -0.79 | [-1.9, 0.24] |  |
| Latino | 0.36 | [-0.54, 1.3] |  | 0.96 | [0.20, 1.7] |  | -0.02 | [-0.80, 0.79] |  | 0.55 | [-0.23, 1.3] |  |
| Multiracial | 1.3 | [-0.11, 2.9] |  | 2.40 | [1.2, 3.8] |  | 1.8 | [0.66, 3.3] |  | 1.1 | [0.21, 2.0] |  |
| Other | -0.46 | [-1.7, 0.77] |  | -0.09 | [-1.2, 0.95] |  | -0.09 | [-1.2, 1.0] |  | 0.62 | [-0.58, 1.7] |  |
| Sexual minority identity | **0.93** | **[0.04, 1.9]** | **0.040** | 0.31 | [-0.56, 1.2] | 0.500 | 0.47 | [-0.38, 1.3] | 0.300 | 0.51 | [-0.40, 1.4] | 0.300 |
| **Public assistance** |  |  | **0.028** |  |  | >0.9 |  |  | 0.400 |  |  | 0.120 |
| Yes vs. No | -1.1 | [-2.1, -0.12] |  | 0.05 | [-0.80, 0.92] |  | -0.37 | [-1.2, 0.50] |  | 0.65 | [-0.18, 1.5] |  |
| Personal income | **0.12** | **[0.02, 0.22]** | **0.022** | -0.08 | [-0.17, 0.00] | 0.062 | 0.04 | [-0.05, 0.13] | 0.400 | -0.04 | [-0.13, 0.05] | 0.400 |
| Urbanicity | -0.51 | [-1.4, 0.37] | 0.300 | -0.66 | [-1.4, 0.09] | 0.083 | -0.52 | [-1.3, 0.26] | 0.200 | -0.45 | [-1.3, 0.35] | 0.300 |
| Alcohol use | -0.14 | [-0.35, 0.06] | 0.200 | -0.07 | [-0.24, 0.10] | 0.400 | -0.02 | [-0.20, 0.16] | 0.800 | 0.15 | [-0.03, 0.33] | 0.095 |
| Drug Use | 0.03 | [-0.04, 0.10] | 0.400 | 0.06 | [0.01, 0.11] | **0.023** | 0.03 | [-0.02, 0.09] | 0.300 | 0.02 | [-0.03, 0.07] | 0.400 |
| Psychiatric distress | **0.19** | **[0.11, 0.28]** | **<0.001** | 0.07 | [0.00, 0.14] | 0.058 | **0.15** | **[0.08, 0.23]** | **<0.001** | **0.08** | **[0.00, 0.15]** | **0.046** |
| Everyday discrimination | 0.05 | [-0.57, 0.66] | 0.900 | 0.16 | [-0.31, 0.63] | 0.500 | 0.17 | [-0.35, 0.69] | 0.500 | **0.8** | **[0.32, 1.3]** | **<0.001** |
| Social wellbeing | **0.57** | **[0.17, 0.99]** | **0.005** | 0.12 | [-0.23, 0.47] | 0.500 | **0.36** | **[0.01, 0.72]** | **0.046** | **0.37** | **[0.00, 0.75]** | **0.049** |
| Healthcare stereotype threat | **0.48** | **[0.08, 0.88]** | **0.017** | **0.6** | **[0.26, 0.96]** | **<0.001** | 0.18 | [-0.16, 0.53] | 0.300 | **0.64** | **[0.29, 1.0]** | **<0.001** |
| Non-affirmation of gender identity | 0.26 | [-0.11, 0.63] | 0.200 | -0.21 | [-0.51, 0.09] | 0.200 | 0.19 | [-0.12, 0.50] | 0.200 | **-0.35** | **[-0.67, -0.03]** | **0.030** |
| Gender identity non-disclosure | -0.25 | [-0.71, 0.19] | 0.300 | 0.00 | [-0.40, 0.39] | >0.9 | -0.16 | [-0.57, 0.25] | 0.400 | -0.03 | [-0.45, 0.40] | 0.900 |
| Internalized transphobia | 0.08 | [-0.33, 0.50] | 0.700 | 0.09 | [-0.25, 0.44] | 0.600 | 0.16 | [-0.19, 0.53] | 0.400 | -0.05 | [-0.40, 0.30] | 0.800 |
| Negative expectations of the future | 0.08 | [-0.46, 0.64] | 0.800 | -0.08 | [-0.52, 0.37] | 0.700 | 0.08 | [-0.38, 0.56] | 0.700 | -0.17 | [-0.65, 0.30] | 0.500 |
| Abbreviations. OR = Odds Ratio, CI = Confidence Interval, GNB = Gender Non-binary | | | | | | | | |  |  |  |  |
| Note. Reference category for gender identity was trans man and for race was White. | | | | | | | | |  |  |  |  |

| **Table 5.** Cox proportional hazards with EMR variables. | | |  |  |  |  |  |  |  |  |  |  |
| --- | --- | --- | --- | --- | --- | --- | --- | --- | --- | --- | --- | --- |
| Characteristics |  | Suicidal Ideation |  | Suicide Intent | | | Suicide Plan | | | Suicide Attempt | | |
|  | HR | 95% CI | p-value | HR | 95% CI | p-value | HR | 95% CI | p-value | HR | 95% CI | p-value |
| **Gender identity** |  |  |  |  |  |  |  |  |  |  |  |  |
| Trans Woman | **0.44** | **[0.26, 0.75]** | **0.002** | 0.54 | [0.26, 1.11] | 0.094 | **0.51** | **[0.30, 0.87]** | **0.013** | **0.20** | **[0.06, 0.65]** | **0.008** |
| GNB | 0.85 | [0.52, 1.39] | 0.500 | 0.95 | [0.48, 1.91] | 0.900 | 0.78 | [0.47, 1.31] | 0.400 | **0.33** | **[0.15, 0.71]** | **0.005** |
| **Race** |  |  |  |  |  |  |  |  |  |  |  |  |
| Black | 1.02 | [0.54, 1.92] | >0.9 | 1.81 | [0.92, 3.56] | 0.087 | 0.94 | [0.49, 1.80] | 0.800 | 1.17 | [0.55, 2.49] | 0.700 |
| Latino | 1.76 | [0.99, 3.12] | 0.056 | **2.54** | **[1.40, 4.61]** | **0.002** | 1.20 | [0.71, 2.01] | 0.500 | 1.60 | [0.60, 4.24] | 0.300 |
| Multiracial | **2.69** | **[1.48, 4.90]** | **0.001** | **4.31** | **[2.16, 8.60]** | **<0.001** | 1.48 | [0.76, 2.88] | 0.200 | 1.81 | [0.57, 5.74] | 0.300 |
| Other | 1.61 | [0.77, 3.36] | 0.200 | 1.08 | [0.31, 3.80] | >0.9 | 0.80 | [0.26, 2.41] | 0.700 | **6.26** | **[1.98, 19.7]** | **0.002** |
| Sexual minority identity | 1.22 | [0.69, 2.14] | 0.500 | 0.91 | [0.47, 1.74] | 0.800 | 1.96 | [0.98, 3.92] | 0.056 | 0.98 | [0.13, 7.38] | >0.9 |
| **Public assistance** |  |  |  |  |  |  |  |  |  |  |  |  |
| Yes vs. No | 1.10 | [0.56, 2.15] | 0.800 | 1.55 | [0.83, 2.90] | 0.200 | **0.44** | **[0.26, 0.73]** | **0.002** | 0.69 | [0.29, 1.66] | 0.400 |
| Abbreviations. HR = Hazard Ratio, CI = Confidence Interval, GNB = Gender Non-binary | | | | | | | | |  |  |  |  |
| Note. Reference category for gender identity was trans man and for race was White. | | | | | | | | |  |  |  |  |

| **Table 6.** Cox proportional hazards with both EMR and psychosocial variables. | | | | | | |  |  |  |  |  |  |
| --- | --- | --- | --- | --- | --- | --- | --- | --- | --- | --- | --- | --- |
| Characteristics |  | Suicidal Ideation |  | Suicide Intent | | | Suicide Plan | | | Suicide Attempt | | |
|  | HR | 95% CI | p-value | HR | 95% CI | p-value | HR | 95% CI | p-value | HR | 95% CI | p-value |
| **Gender identity** |  |  |  |  |  |  |  |  |  |  |  |  |
| Trans Woman | **0.38** | **[0.21, 0.69]** | **0.002** | 1.03 | [0.42, 2.55] | >0.9 | **0.48** | **[0.27, 0.85]** | **0.012** | 0.14 | [0.01, 1.72] | 0.120 |
| GNB | 0.86 | [0.51, 1.47] | 0.600 | 1.69 | [0.58, 4.91] | 0.300 | 1.4 | [0.77, 2.56] | 0.300 | 0.35 | [0.06, 1.84] | 0.200 |
| **Race** |  |  |  |  |  |  |  |  |  |  |  |  |
| Black | 1.71 | [0.89, 3.30] | 0.110 | 0.88 | [0.34, 2.27] | 0.800 | 0.88 | [0.43, 1.81] | 0.700 | 1.52 | [0.33, 6.86] | 0.600 |
| Latino | **3.21** | **[1.96, 5.25]** | **<0.001** | **2.25** | **[1.15, 4.38]** | **0.018** | 1.49 | [0.93, 2.38] | 0.100 | 2.70 | [0.93, 7.82] | 0.067 |
| Multiracial | **3.11** | **[1.52, 6.33]** | **0.002** | 0.63 | [0.30, 1.31] | 0.200 | 1.09 | [0.56, 2.15] | 0.800 | 1.67 | [0.23, 12.1] | 0.600 |
| Other | **3.02** | **[1.20, 7.63]** | **0.019** | 1.13 | [0.30, 4.34] | 0.900 | **3.47** | **[1.64, 7.35]** | **0.001** | **9.72** | **[1.13, 83.7]** | **0.038** |
| Sexual minority identity | 1.33 | [0.51, 3.52] | 0.600 | 0.94 | [0.31, 2.81] | >0.9 | 1.83 | [0.74, 4.54] | 0.200 | 0.21 | [0.00, 12.4] | 0.500 |
| **Public assistance** |  |  |  |  |  |  |  |  |  |  |  |  |
| Yes vs. No | 0.58 | [0.23, 1.47] | 0.200 | **0.23** | **[0.09, 0.58]** | **0.002** | **0.32** | **[0.17, 0.60]** | **<0.001** | 0.5 | [0.05, 5.59] | 0.600 |
| Personal Income | 0.96 | [0.88, 1.05] | 0.400 | 0.96 | [0.88, 1.04] | 0.300 | 0.98 | [0.90, 1.06] | 0.600 | 1.07 | [0.85, 1.34] | 0.600 |
| Urbanicity | **0.50** | **[0.31, 0.81]** | **0.005** | 1.87 | [0.73, 4.77] | 0.200 | 1.08 | [0.67, 1.73] | 0.800 | 1.07 | [0.31, 3.67] | >0.9 |
| Alcohol use | **0.80** | **[0.71, 0.91]** | **<0.001** | 0.85 | [0.71, 1.01] | 0.063 | 1.03 | [0.92, 1.16] | 0.600 | 0.83 | [0.53, 1.30] | 0.400 |
| Drug Use | 1.03 | [1.00, 1.06] | 0.087 | 0.99 | [0.96, 1.02] | 0.500 | **0.93** | **[0.90, 0.97]** | **<0.001** | 0.99 | [0.93, 1.06] | 0.800 |
| Psychiatric distress | **1.07** | **[1.02, 1.12]** | **0.009** | **1.14** | **[1.05, 1.25]** | **0.003** | **1.10** | **[1.05, 1.15]** | **<0.001** | 0.99 | [0.83, 1.20] | >0.9 |
| Everyday discrimination | 1.35 | [0.96, 1.91] | 0.089 | **2.04** | **[1.22, 3.41]** | **0.007** | **1.44** | **[1.05, 1.97]** | **0.023** | 1.32 | [0.67, 2.62] | 0.400 |
| Social wellbeing | 1.00 | [0.82, 1.23] | >0.9 | **1.56** | **[1.08, 2.26]** | **0.018** | 1.06 | [0.81, 1.38] | 0.700 | 0.77 | [0.36, 1.65] | 0.500 |
| Healthcare stereotype threat | 1.11 | [0.79, 1.55] | 0.500 | 0.97 | [0.63, 1.50] | 0.900 | 1.23 | [0.89, 1.70] | 0.200 | 1.60 | [0.28, 9.00] | 0.600 |
| Non-affirmation of gender identity | 0.88 | [0.67, 1.17] | 0.400 | 0.78 | [0.60, 1.03] | 0.080 | 0.81 | [0.62, 1.06] | 0.130 | 1.33 | [0.71, 2.47] | 0.400 |
| Gender identity non-disclosure | 1.23 | [0.95, 1.59] | 0.110 | 1.09 | [0.79, 1.52] | 0.600 | **1.40** | **[1.09, 1.79]** | **0.008** | 1.88 | [0.54, 6.57] | 0.300 |
| Internalized transphobia | **0.71** | **[0.54, 0.93]** | **0.013** | 1.11 | [0.82, 1.51] | 0.500 | 0.87 | [0.66, 1.16] | 0.300 | 0.91 | [0.28, 3.02] | 0.900 |
| Negative expectations of the future | 1.01 | [0.74, 1.37] | >0.9 | 1.06 | [0.70, 1.60] | 0.800 | 0.87 | [0.63, 1.20] | 0.400 | 0.97 | [0.47, 1.99] | >0.9 |
| Abbreviations. HR = Hazard Ratio, CI = Confidence Interval, GNB = Gender Non-Binary | | | | | | | |  |  |  |  |  |
| Note. Reference category for gender identity was trans man and for race was White. | | | | | | | | |  |  |  |  |

**Table 7.** *Model performance.*

|  | **Models with Ordinal Outcomes** | | | | | | | | **Models with Age of Onset Outcomes (RMSE)** | |  |
| --- | --- | --- | --- | --- | --- | --- | --- | --- | --- | --- | --- |
|  | **Tree Model Accuracy** | **Logistic Accuracy** | **Logistic AUC (Never vs Once + More Than Once)** | **Logistic AUC (Never + Once vs More Than Once)** | **Logistic AUC (Once vs Never + More Than Once)** | **Tree AUC (Never vs Once + More Than Once)** | **Tree AUC (Never + Once vs More Than Once)** | **Tree AUC (Once vs Never + More Than Once)** | **Tree RMSE** | **Cox Proportional Hazards RMSE** | |
| **Analyses with EMR variables** | | | | | | | | | | |  |
| Suicidal Ideation | 0.604 | 0.663 | 0.7802 | 0.7297 | 0.5811 | 0.7523 | 0.7065 | 0.5878 | 9.04 | 17.53 | |
| Suicide Intent | 0.476 | 0.5333 | 0.6893 | 0.6676 | 0.6044 | 0.5282 | 0.543 | 0.5194 | 5.734 | 16.42 | |
| Suicide Plan | 0.55 | 0.5519 | 0.6822 | 0.6745 | 0.5551 | 0.6376 | 0.6443 | 0.5343 | 8.739 | 18.349 | |
| Suicide Attempt | 0.637 | 0.6704 | 0.7207 | 0.7587 | 0.5793 | 0.5 | 0.5 | 0.5 | 7.24 | 18.776 | |
| **Analyses with EMR and psychosocial variables** | | | | | | | | | | |  |
| Suicidal Ideation | 0.608 | 0.6963 | 0.8359 | 0.809 | 0.659 | 0.7069 | 0.6901 | 0.5812 | 9.04 | 18.279 | |
| Suicide Intent | 0.568 | 0.5889 | 0.7316 | 0.7516 | 0.5461 | 0.6735 | 0.6757 | 0.5362 | 5.734 | 16.543 | |
| Suicide Plan | 0.604 | 0.5963 | 0.7566 | 0.7502 | 0.6186 | 0.7042 | 0.717 | 0.5328 | 8.24 | 18.411 | |
| Suicide Attempt | 0.685 | 0.6852 | 0.7676 | 0.8518 | 0.5551 | 0.6213 | 0.6624 | 0.5214 | 7.24 | 20.321 | |

Abbreviations. AUC = Area under the curve; RMSE = root mean squared error
